# The Role of Creative Mindsets in the Relationship Between Metacognitive Experience and Divergent Thinking: A Metacognitive Perspective

**DOI:** 10.3390/jintelligence13030027

**Published:** 2025-02-24

**Authors:** Xiaoyu Jia, Ping Li, Weijian Li

**Affiliations:** 1 School of Psychology, Zhejiang Normal University, Jinhua 321000, China; jiaxiaoyu@126.com; 2 College of Teacher Education, Southwest University, Chongqing 400715, China; 3 School of Education Science, Guangdong Polytechnic Normal University, Guangzhou 510000, China; liping_psy@gpnu.edu.cn

**Keywords:** creative mindsets, metacognitive experience, retrieval fluency, cognitive persistence, alternative uses task

## Abstract

Metacognition is vital for creativity; however, the specific contributions of its components (i.e., metacognition knowledge, metacognition experience, and metacognition monitoring and control) have received varying levels of attention, particularly due to the limited research on metacognitive experience. Additionally, the interactions among these components in influencing creative cognition remain unclear. We conducted two experiments to explore the influence of metacognitive experience on divergent thinking (e.g., alternative uses tasks, AUT) and the moderating role of creative mindsets—a core element of metacognitive knowledge—in this process. In Experiment 1, retrieval fluency, measured by the quantity of the ideas generated, was used to activate varying levels of metacognitive experience (fluency vs. disfluency) during the AUT. The findings showed that the originality of ideas generated under the disfluency condition was significantly higher than under the fluency condition, suggesting a positive effect of metacognitive disfluency experience on AUT. In Experiment 2, a multiple-choice task was used to prime individuals’ creative mindsets (entity vs. incremental). The results indicated that individuals with a creative growth mindset exhibited greater cognitive persistence under the disfluency condition, subsequently enhancing the originality of their ideas, indicating that creative mindsets moderate the effect of metacognitive disfluency experience on AUT performance via cognitive persistence. We integrated previous findings to describe the interactive impacts of creative mindsets, metacognitive experience, and metacognitive monitoring and control on divergent and convergent creative thinking processes within a metacognitive framework, providing a model to reveal the dynamic interplay of metacognitive processes in creative cognition. Practically, fostering individuals’ growth-oriented creative mindsets may represent a promising avenue for creativity development.

## 1. Introduction

Creative thinking is generally defined as the ability to come up with ideas that are both novel and appropriate (Sternberg and Lubart 1991). In recent years, the critical role of metacognition in creative thinking has gained increasing attention (Erbas and Bas 2015; Jia et al. 2019, 2023; Karwowski et al. 2020; Lebuda and Benedek 2023; Pesut 1990; Puente-Díaz et al. 2021; Puryear 2015). Metacognition is a complex system that includes metacognitive knowledge, experience, and monitoring and control (Efklides 2008; Flavell 1976). Previous studies have predominantly focused on the influence of metacognitive knowledge (e.g., creative beliefs, Beghetto et al. 2021; Royston and Reiter-Palmon 2019) and metacognitive monitoring and control (e.g., conflict monitoring and strategy switching, Jia et al. 2022) on creative thinking. However, metacognitive experience has received limited attention in creativity research (Puente-Diaz and Cavazos-Arroyo 2022). As a source of information for the creative process, metacognitive experience could provide available cues for individuals to rely on when generating or evaluating the creativity of their own ideas (Koriat 2008; Puente-Díaz 2023). Considering that it is closely related to creative self-beliefs (Puente-Diaz and Cavazos-Arroyo 2022) and the monitoring state for creative idea outputs (Preiss 2022; Puente-Díaz et al. 2021), further exploration of whether and how metacognitive experience, including metacognitive (dis)fluency experience, affects creativity is of great significance in revealing the metacognitive attributes of creativity.

### 1.1. Effect of Metacognitive (Dis)fluency Experience on Divergent Thinking

Metacognitive (dis)fluency experience, which refers to the subjective perception of the ease or difficulty of cognitive operations, can be indicated by a metacognitive cue of processing fluency (Koriat et al. 2004). The ease-of-processing hypothesis (Koriat 2008) posits that processing fluency influences processing styles (Alter et al. 2007), cognitive persistence (Jia et al. 2022; Toyama et al. 2023), and strategy selection (Lucas and Nordgren 2015)—all critical factors in creativity (Gilhooly et al. 2007). Empirical findings suggest that metacognitive (dis)fluency experience can inform the degree of creativity of the ideas generated (Mehta et al. 2012; Puente-Díaz 2023; Puente-Diaz and Cavazos-Arroyo 2020). For example, Mehta et al. (2012) found that experiences of processing disfluency, triggered by moderate noise, fostered more constructive or abstract thinking, thereby enhancing the originality of ideas in the divergent thinking task. Additionally, Puente-Diaz and Cavazos-Arroyo (2020) asked participants to recall either two or six examples from their lives in which they generated creative ideas that solved a problem or improved something, subsequently tasking them with generating ideas to improve smartphones. The findings indicated that pre-task metacognitive (dis)fluency experience, as reflected by the recall assessment, led to higher creativity. Although these studies highlight the relationship between metacognitive (dis)fluency experience and creativity, one limitation that cannot be ignored is that metacognitive (dis)fluency experiences were elicited through indirect manipulations. Consequently, metacognitive (dis)fluency experiences triggered by environmental variables, such as noise or background music, can be significantly influenced by factors beyond the experimental context (Mehta et al. 2012; Threadgold et al. 2019). Moreover, compared to metacognitive (dis)fluency experiences arising from the actual process of divergent thinking, those induced by prior tasks may have a weaker impact on idea selection and innovation in divergent thinking tasks (Puente-Diaz and Cavazos-Arroyo 2020). Therefore, directly manipulating participants’ metacognitive (dis)fluency experiences during creative tasks—rather than relying on indirect methods—has been deemed necessary by researchers (Jia et al. 2023; Puente-Díaz 2023; Puente-Diaz and Cavazos-Arroyo 2020). For example, Jia et al. (2023) used font-style manipulation to directly manipulate participants’ perceptual fluency during the Chinese logogriph task, an insight problem that reflects convergent thinking (Qiu et al. 2010). The results showed a negative effect of metacognitive disfluency experience on logogriph-solving performance.

Although metacognitive disfluency experiences can serve as important cues for individuals when conducting creative operations, their diagnosticity varies depending on the type of creative problems being solved. Unlike convergent thinking tasks, which have a single, correct answers, divergent thinking tasks—lacking a definitive solution—may render cues less valid, although they remain crucial for the potential originality of the ideas generated (Puente-Díaz 2023). Along with the distinct cognitive processing mechanism involved in divergent and convergent thinking tasks (Benedek et al. 2011) and the idea that metacognitive dynamics exhibit different characteristics when addressing these two types of creative tasks (Puente-Díaz et al. 2021), this study aims to directly manipulate individuals’ metacognitive (dis)fluency experiences during divergent thinking tasks (e.g., the alternative uses task, AUT) to assess their impact on creative idea generation.

Retrieval fluency, a type of processing fluency, is commonly used to reflect metacognitive experience. The main idea is to vary the number of specific events that participants are asked to retrieve (Murphy and Castel 2021; Sanna et al. 2002; Schwarz et al. 1991). For example, Sanna et al. (2002) found that increasing retrieval difficulty by asking participants to list more supporting ideas could trigger participants’ experience of disfluency. Based on this, we aimed to create different levels of retrieval fluency by controlling the number of ideas generated by participants in our study, thus examining its impact on AUT performance. Unlike the typical evaluation of creative ability from the three aspects of fluency (i.e., the number of valid ideas generated), originality (i.e., the novelty of the ideas generated), and flexibility (i.e., the variety of different types of ideas generated) (De Dreu et al. 2008; Guilford 1967), this study uses the average originality of ideas, calculated by dividing the originality score by the fluency score, as the core indicator of creativity (Benedek et al. 2013; Silvia et al. 2008). Since manipulating the number of ideas in the experiment directly affects the fluency score, and considering that the quality of ideas sometimes better reflects an individual’s creativity level than quantity (Batey and Furnham 2008; Reiter-Palmon and Arreola 2015), the average originality of ideas is emphasized.

A series of behavioral and neuroimaging studies have shown that varying degrees of metacognitive fluency experiences can trigger different cognitive processing mechanisms. Specifically, experience of metacognitive fluency tends to engage intuitive processing, whereas experience of metacognitive disfluency is more likely to evoke analytical processing (Alter et al. 2007; Murphy et al. 2022; Unkelbach and Greifeneder 2013). In line with dual-process models of creativity (Nijstad et al. 2010), which propose that creativity results from the interaction of intuitive processes and deliberate analytical thought, prior research has indicated that moderate levels of noise-induced metacognitive disfluency experience can enhance performance on divergent thinking tasks (Mehta et al. 2012). Based on these insights, we propose that different levels of metacognitive (dis)fluency, created by manipulating the number of ideas participants were required to generate for each object, may influence the originality of ideas in the AUT. That is, the originality of ideas under the metacognitive disfluency experience condition will be significantly higher than under the metacognitive fluency experience condition when completing AUT.

### 1.2. Possible Role of Creative Mindsets in the Relationship Between Metacognitive (Dis)fluency Experiences and Divergent Thinking

Although many variables can affect an individual’s experience of metacognitive fluency, how individuals interpret this experience, or what kind of information they infer from it, depends on the theoretical beliefs they hold (Schwarz 2004). Previous studies have demonstrated that whether individuals believe abilities are fixed or malleable influences their interpretation of metacognitive fluency experiences (Jia et al. 2023; Labroo and Kim 2009). The main idea is that individuals with a growth belief might interpret metacognitive disfluency experience as a lack of effort, whereas those with an entity belief might view it as a deficiency in ability. This different interpretation for metacognitive (dis)fluency experiences based on the two types of ability beliefs, in turn, modulates the relationship between metacognitive (dis)fluency experiences and various cognitive performances, such as reading comprehension (Miele and Molden 2010) and learning achievement (Blackwell et al. 2007), as well as metacognitive judgments of learning (Molden and Dweck 2006).

Since beliefs about ability can vary in generality and specificity across different domains (Zhu et al. 2020), Karwowski (2014) introduced the concept of creative mindsets, referring specifically to an individuals’ beliefs about their creative ability. Those who hold a growth view of creativity believe that it can be improved through effort, whereas those with a fixed view regard creativity as innate and unchangeable (Karwowski et al. 2019). Studies have shown that individuals with a creative growth mindset tend to exhibit higher levels of creative self-efficacy, creative personality, creative achievement, and problem-solving abilities (Hass et al. 2016; O’Connor et al. 2013; Redifer et al. 2019; Royston and Reiter-Palmon 2019; Zhou et al. 2020). Similar to how individuals with growth or fixed mindsets differ in their sensitivity to metacognitive (dis)fluency experiences and in their use of those (dis)fluency experiences to guide idea selection during divergent thinking tasks (Betsch and Roth 2018; Puente-Díaz et al. 2021), this study aims to further explore how individuals with different creative mindsets perform on divergent thinking tasks under varying metacognitive fluency experience conditions.

Puente-Díaz (2023) proposed a two-way interaction between creative self-beliefs and metacognitive feelings. While previous studies revealed that metacognitive feelings can serve as cues for creative self-beliefs, such as self-efficacy in idea-generation tasks (Puente-Diaz and Cavazos-Arroyo 2020), the antecedent role of creative self-beliefs (e.g, creative mindsets) in shaping metacognitive disfluency feelings warrants further exploration (Puente-Díaz 2023). Jia et al. (2023) activated individuals’ creative mindsets through a multiple-choice priming method (O’Connor et al. 2013) in a Chinese logogriph-solving task. The results indicated that compared to individuals with a creative fixed mindset, those with a creative growth mindset interpreted metacognitive disfluency experience as a lack of effort. Consequently, they exhibited greater cognitive persistence to compensate for the negative effect of processing disfluency on logogriphs solving, consistent with findings from previous studies on learning (Blackwell et al. 2007; Jia et al. 2016; Miele and Molden 2010; Undorf et al. 2017). According to the dual pathway creativity model, creativity can be achieved through two routes: cognitive persistence and cognitive flexibility (De Dreu et al. 2008; Nijstad et al. 2010). Specifically, the generation of creative ideas is often hindered by individuals’ knowledge of conventional uses, and only by discarding these constraints and applying persistence, prolonged effort, and deep exploration of a few cognitive categories can genuinely creative ideas emerge. Additionally, it can also be achieved through breaking set patterns and thinking flexibly to utilize a broad range of cognitive categories (Baas et al. 2011; Baas et al. 2015; Gilhooly et al. 2007; Lucas and Nordgren 2015; Wu and Koutstaal 2022; Toyama et al. 2023). Given the differentiated interpretation of metacognitive (dis)fluency experiences by individuals with different creative mindsets (Molden and Dweck 2006), along with the significant correlation between creative growth mindset and cognitive persistence (Jia et al. 2023; Puente-Diaz and Cavazos-Arroyo 2022), we hypothesize that metacognitive disfluency experience will elicit stronger cognitive persistence, rather than cognitive flexibility, in individuals with a creative growth mindset, thereby improving their idea-generation performance.

### 1.3. The Present Study

This study aims to utilize the retrieval fluency paradigm, manipulating the number of ideas generated by participants during AUT (Sanna et al. 2002), to induce varying levels of metacognitive (dis)fluency experiences and examine their impact on idea originality. Additionally, multiple-choice priming (O’Connor et al. 2013) was used to activate different types of situational creative mindset, further exploring the role of creative mindsets in the relationship between metacognitive (dis)fluency experiences and the originality of ideas in the AUT. The hypotheses are as follows:

**Hypothesis** **1.** *There would be relationships between metacognitive (dis)fluency experiences and AUT performance. Individuals’ metacognitive disfluency experience could enhance the average originality of their ideas, whereas individuals’ metacognitive fluency experience could diminish the average originality of their ideas.*

**Hypothesis** **2.** *Individual creative mindsets might play moderating roles in the relationship between metacognitive (dis)fluency experiences and AUT performance. Compared with individuals with a creative fixed mindset, those with a creative growth mindset would demonstrate greater cognitive persistence in the metacognitive disfluency experience condition, resulting in higher average originality of ideas in the AUT.*

## 2. Experiment 1

This study primarily examines the impact of metacognitive (dis)fluency experiences on AUT performance. Taking the characteristics of AUT into account, we manipulated retrieval fluency, which is measured by the quantity of ideas generated, to activate different levels of metacognitive (dis)fluency experiences (fluency and disfluency experiences, Sanna et al. 2002). We hypothesize that AUT performance under the metacognitive disfluency experience condition will be significantly higher than under the metacognitive fluency experience condition.

### 2.1. Methods

#### 2.1.1. Participants

We used G*Power 3.1 software to determine the minimum sample size required for an effect size of 0.25, an alpha level of 0.05, and a power of 0.80 (Faul et al. 2007). The expected sample size was 128. A total of 143 university students were recruited online to participate in the experiment for compensation. Data from 5 participants were excluded due to incomplete responses, resulting in a final sample of 138 participants (*M* = 19.03, *SD* = 0.81), consisting of 60 males and 78 females. None of the participants had previously taken part in similar experiments. The study was ethically approved by the Research Ethics Committee of Southwest University.

#### 2.1.2. Task and Design

The alternative uses task (AUT, Guilford 1967) was used to assess participants’ divergent thinking performance. Participants were instructed to generate predetermined unusual uses for a brick, a cardboard box, and an umbrella within a 5 min timeframe for each object. A between-subjects design was used, with task requirement (i.e., the number of ideas participants were asked to generate) as the independent variable, which had 2 levels: “think of 7 novel and unique uses for each object within 5 min” (abbreviated as “7 ideas”) and “think of 14 or more novel and unique uses for each object within 5 min” (abbreviated as “14 or more ideas”). The dependent variable was the average originality of the ideas in the AUT.

#### 2.1.3. Metacognitive (Dis)fluency Experience Manipulation

In a pilot study, 141 participants were asked to generate novel and unique uses for a brick, a cardboard box, and an umbrella within 5 min for each object. The results indicated that participants generated an average of 20.62 ideas over the total 15 min (approximately 21 ideas), which equates to an average of 7 ideas every 5 min. Accordingly, generating 7 ideas in 5 min was used as a baseline to activate different levels of metacognitive (dis)fluency experiences among participants. Specifically, “think of 7 novel and unique uses for each object within 5 min” served as the manipulation for the metacognitive fluency experience, while “think of 14 or more novel and unique uses for each object within 5 min” served as the manipulation for the metacognitive disfluency experience. After the AUT, we evaluated the effectiveness of the metacognitive (dis)fluency experience manipulation by asking participants to rate their degree of retrieval fluency when generating ideas on a 7-point scale, ranging from “1 = high disfluency” to “7 = high fluency”, which aligns with previous research (Sanna et al. 2002; Schwarz et al. 1991).

#### 2.1.4. Processing Motivation Questionnaire

Similar to Mehta et al. (2012), participants’ processing motivation was evaluated using three questions: “To what extent are you willing to complete this task?”, “To what extent do you enjoy engaging in this task?”, and “How interesting do you find this task?” Participants rated their responses to each question on a 7-point scale, ranging from “1 = not at all” to “7 = very much”, with the total scores of these three questions indicating their processing motivation”.

#### 2.1.5. Procedure

Participants completed the experiment individually on a computer. Instructions for the AUT were presented in PowerPoint format, with half of the participants receiving the instruction: “Please think of 7 novel and unique uses for each object within 5 min”, while the other half received the instruction: “Please think of 14 or more of novel and unique uses for each object within 5 min”. The types of instructions were randomly assigned among participants. A computer recorded and reminded participants of their remaining response time. After completing the AUT, participants were required to answer questions regarding their metacognitive (dis)fluency experiences and processing motivation evaluations.

### 2.2. Results

#### 2.2.1. Manipulation Check

A manipulation check was conducted to evaluate the effectiveness of the metacognitive (dis)fluency experience manipulation. An independent samples t-test revealed that the metacognitive fluency experience induced by the “7 ideas” condition (*M* = 4.83, *SD* = 1.60) was significantly higher than that induced by the “14 or more ideas” condition (*M* = 3.76, *SD* = 1.31), *t* (136) = 4.29, *p* < 0.001, Cohen’s *d* = 0.73, indicating that the metacognitive (dis)fluency experience manipulation was effective.

#### 2.2.2. Processing Motivation

The total score of each participant responses to the three motivation questions was examined to test whether the number of ideas generated changed participants’ processing motivation. An independent samples *t*-test showed that there was no significant difference in motivation levels between the “7 ideas” condition (*M* = 9.81, *SD* = 3.07) and the “14 or more ideas” condition (*M* = 9.56, *SD* = 2.24), *t* (136) = 0.54, *p* = 0.58.

#### 2.2.3. Originality of Ideas

Three independent raters evaluated participants’ responses to three items based on fluency, originality, and flexibility (Guilford 1967). The inter-rater reliability across the three raters ranged from 0.89 to 0.95. The average originality of ideas generated, calculated by dividing the originality score by the fluency score, was used as the core index to reflect individuals’ creativity (Benedek et al. 2013; Silvia et al. 2008). An independent samples t-test showed that the average originality of ideas under the “14 or more ideas” condition (*M* = 2.65, *SD* = 0.27) was significantly higher than under the “7 ideas” condition (*M* = 2.50, *SD* = 0.36), *t* (136) = 2.69, *p* < 0.05, *Cohen’s d* = 0.47.

### 2.3. Discussion

Experiment 1 found that the average originality of ideas generated under the metacognitive disfluency experience condition was significantly higher than those under the metacognitive fluency experience condition, indicating that metacognitive (dis)fluency experiences, as reflected by retrieval fluency, impact divergent thinking performance. According to the notion that metacognitive fluency experience prompts individuals to use intuitive and effortless fast processing, while metacognitive disfluency experience triggers more analytical processing (Alter and Oppenheimer 2009; Eitel et al. 2014; Eitel and Kühl 2016; Murphy et al. 2022), the positive effect of analytical processing on divergent thinking performance is consistent with previous studies (George and Zhou 2007; Mehta et al. 2012).

## 3. Experiment 2

The purpose of this study is to examine the effects of individuals’ creative mindsets and metacognitive (dis)fluency experiences on divergent thinking performance, as well as the role of cognitive persistence in this relationship. A multiple-choice task was used to prime the creative mindsets (entity vs. incremental) among participants (O’Connor et al. 2013). We hypothesize that creative mindsets moderate the mediating effect of cognitive persistence in the relationship between metacognitive (dis)fluency experiences and divergent thinking performance.

### 3.1. Methods

#### 3.1.1. Participants

A power analysis using G*Power 3.1 was conducted to determine the minimum sample size required for an effect size of 0.25, an alpha level of 0.05, and a power of 0.80. The expected sample size was 179. A total of 215 university students were recruited online to participate in this experiment for compensation. Data from 1 participant were excluded due to incomplete questionnaire responses, resulting in a final valid sample of 214 participants (*M* = 19.76, *SD* = 0.94), which included 57 males and 157 females. None of the participants had previously participated in similar experiments. These participants were randomly assigned to four groups.

#### 3.1.2. Task and Design

The AUT followed the same procedure as Experiment 1, with two exceptions: (1) the number of objects was reduced from three to two, as participants were instructed to generate predetermined unusual uses for a cardboard box and an umbrella; (2) the response time for each object was changed from five to three minutes. Since priming the creative mindsets prior to the AUT requires time, both adjustments aimed to reduce potential participant fatigue. A 2 (creative mindset: fixed or growth) × 2 (task requirement: 5 ideas, 10 or more ideas) between-subjects design was used, with the dependent variable being the average originality of ideas in the AUT.

#### 3.1.3. Creative Mindset Manipulation

An adapted multiple-choice task was employed to prime participants’ creative mindsets (Jia et al. 2023; O’Connor et al. 2013). The task consisted of eight multiple-choice questions, of which the first six were unrelated to creativity. For example, one of the questions presented the following quote: “The one who knows others is wise, but the one who knows themselves is enlightened. The one who overcomes others has strength, but the one who overcomes themselves is truly powerful”. The last two questions were specifically designed to activate distinct creativity mindsets (growth mindset vs. fixed mindset). In the creative fixed mindset manipulation condition, the two quotations emphasized the idea that creativity is inherited and immutable. For example, one quotation stated: “I believe that the most creative individuals are exceptional, born with qualities that most of us lack and will never possess”. In contrast, the creative growth mindset manipulation condition conveyed the idea that creativity is malleable and adaptable. One such quotation stated: “I don’t believe the most creative individuals are born exceptional, they simply strive to cultivate qualities that most of us have yet to develop”.

Each quotation was displayed in PowerPoint format, and participants were required to select an answer from options (A, e.g., “Steve Jobs”; B, e.g., “Socrates”; or C, e.g., “Lao Tzu”) within a 20 s time limit. After making their selection, participants received immediate feedback on the correct answer after 5 s. It is important to note that the accuracy of participants’ responses was not relevant to the study’s purpose, and, therefore, response accuracy was not analyzed in the results.

#### 3.1.4. Metacognitive (Dis)fluency Experience Manipulation

We established a baseline of five ideas generated in three minutes to activate different levels of metacognitive (dis)fluency experiences. Specifically, “think of 5 novel and unique uses for each object within 3 min” (abbreviated as “5 ideas”) served as the manipulation for the metacognitive fluency experience, while “think of 10 or more of novel and unique uses for each object within 3 min” (abbreviated as “10 or more ideas”) served as the manipulation for the metacognitive disfluency experience. After completing the AUT, we evaluated the effectiveness of the processing fluency manipulation. Participants were asked to rate their degree of retrieval fluency when generating ideas on a 7- point scale, ranging from “1 = high disfluency” to “7 = high fluency”.

#### 3.1.5. Manipulation Checks

We evaluated the effectiveness of the metacognitive (dis)fluency experience and creative mindset manipulations. The metacognitive (dis)fluency experience manipulation check was consistent with Experiment 1. To assess the creative mindset manipulation, we employed two methods, as described by Jia et al. (2023). First, participants were asked which of the following statements about creativity they agree with: (A) an individual’s creativity is inherently stable and difficult to improve through effort; (B) an individual’s creativity is malleable and can be improved through effort. Second, participants completed an adapted three-item implicit theory of creativity scale (Dweck and Henderson 1988), with “intelligence” replaced by “creativity”. Higher scores indicated a stronger creative fixed mindset.

#### 3.1.6. Procedure

Each participant was tested individually on a computer. They were asked to complete a multiple-choice task, followed by the AUT, processing fluency manipulation questions and creative mindset manipulation questions in sequence.

### 3.2. Results

#### 3.2.1. Manipulation Checks

An independent samples t-test was conducted to examine the effectiveness of the metacognitive (dis)fluency experience manipulation. The results showed that the metacognitive fluency experience induced by the “5 ideas” condition (*M* = 4.03, *SD* = 1.33) was significantly higher than that induced by the “10 or more ideas” condition (*M* = 2.97, *SD* = 1.47), *t* (212) = 5.46, *p* < 0.001, Cohen’s *d* = 0.76, suggesting that the manipulation of different levels of metacognitive (dis)fluency experience induced by varying the number of ideas required was effective.

Two methods were used to assess the effectiveness of the creativity mindset manipulation. Similarly to Miele and Molden (2010), an independent samples t-test was conducted on the creativity mindset questionnaire scores for the two types of participants. The results showed that participants primed for the creative growth mindset (*M* = 8.50, *SD* = 3.54) scored significantly lower than those primed for the creative fixed mindset (*M* = 9.48, *SD* = 3.41), *t* (212) = 2.03, *p* < 0.05, Cohen’s *d* = 0.28.

Additionally, a chi-square test was conducted to compare the number of participants with consistent choices (where the prime condition and choice result were aligned) versus those with inconsistent choices (where the prime condition and choice result were not aligned) under the creative growth mindset prime condition (consistent number: 64) and the creative fixed mindset prime condition (consistent number: 102). The results showed a significant difference, χ^2^ = 41.12, *p* < 0.001. These findings indicated that the manipulation of creativity mindsets was effective.

Three independent raters independently rated participants’ responses to items on fluency, originality, and flexibility. The results showed an inter-rater reliability of 0.91 to 0.99. The final scores for each participant on fluency, originality, and flexibility were averaged across the three raters’ scores.

#### 3.2.2. Originality of Ideas

The average originality of the ideas generated was used to reflect AUT performance, similar to experiment 1. A 2 (creative mindset: fixed or growth) × 2 (task requirement: 5 ideas, 10 or more ideas) ANOVA revealed a significant main effect of creative mindset, *F*(1, 210) = 18.84, *p* < 0.001, η_P_^2^ = 0.08, indicating that the average originality was higher for the participants primed for the creative growth mindset (*M* = 2.22, *SD* = 0.04) than for those primed for the creative fixed mindset condition (*M* = 2.09, *SD* = 0.03). The main effect of task requirement was not significant, *F*(1, 210) = 1.14, *p* = 0.29, η_P_^2^ = 0.01. Moreover, the interaction between creative mindset and task requirement was significant, *F*(1, 210) = 9.38, *p* < 0.01, η_P_^2^ = 0.04. Specifically, the average originality was higher in the “10 or more ideas” condition (*M* = 3.01, *SD* = 0.54) than in the “5 ideas” condition (*M* = 2.78, *SD* = 0.36) for individuals primed for the creative growth mindset *F*(1, 213) = 8.30, *p* < 0.001, η_P_^2^ = 5.47, whereas there was no significant difference in the average originality between the “10 or more ideas” condition (*M* = 2.59, *SD* = 0.36) and the “5 ideas” condition (*M* = 2.71, *SD* = 0.35) for individuals primed for the creative fixed mindset, *F*(1, 213) = 2.77, *p* = 0.10.

#### 3.2.3. Cognitive Flexibility and Cognitive Persistence

Cognitive flexibility is defined as the variety of ideas generated, reflecting an individual’s flexibility score of tasks (De Dreu et al. 2008). A 2 (creative mindset: fixed or growth) × 2 (task requirement: 5 ideas, 10 or more ideas) ANOVA revealed a marginally significant main effect of creative mindset, *F*(1, 210) = 3.40, *p* = 0.07, η_P_^2^ = 0.02, indicating that the cognitive flexibility was higher for the participants primed for the creative growth mindset (*M* = 2.80, *SD* = 0.07) than for those primed for the creative fixed mindset condition (*M* = 2.64, *SD* = 0.06). Additionally, the main effect of task requirement was significant, *F*(1, 210) = 44.11, *p* < 0.001, η_P_^2^ = 0.17, indicating that the cognitive flexibility was higher under the “10 or more ideas” condition (*M* = 3.01, *SD* = 0.06) than under the “5 ideas” condition (*M* = 2.43, *SD* = 0.06). However, the interaction between creative mindset and task requirement was not significant, *F*(1, 210) = 0.92, *p* = 0.34.

**Figure 1:**
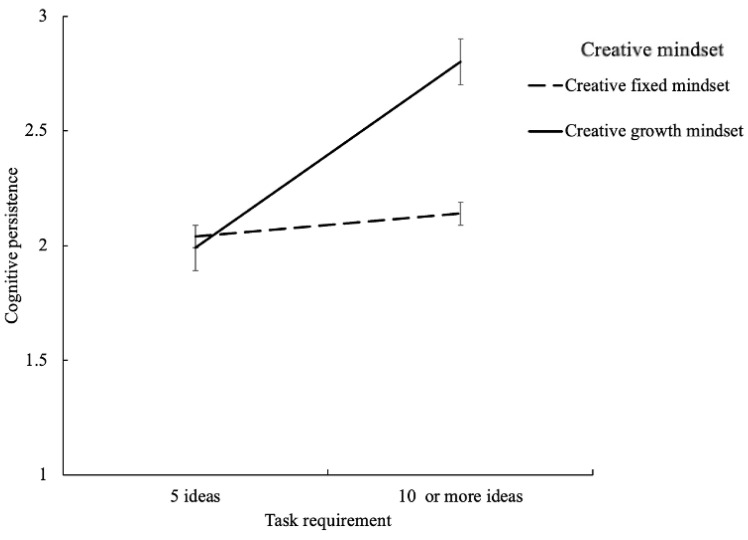
The moderated role of creative mindset in the metacognitive (dis)fluency experience–cognitive persistence relationship.

Cognitive persistence can be calculated through intra-category fluency, as described by De Dreu et al. (2008). This process involves first categorizing all the ideas (flexibility score), then counting the number of ideas generated within each category, and finally averaging these counts to obtain the cognitive persistence score. A 2 (creative mindset: fixed or growth) × 2 (task requirement: 5 ideas, 10 or more ideas) ANOVA (Figure 1) revealed a significant main effect of creative mindset, *F*(1, 210) = 16.30, *p* < 0.001, η_P_^2^ = 0.07, indicating that cognitive persistence was higher for the participants primed for the creative growth mindset (*M* = 2.43, *SD* = 0.81) than for those primed for the creative fixed mindset condition (*M* = 2.09, *SD* = 0.40). Additionally, the main effect of task requirement was significant, *F*(1, 210) = 36.45, *p* < 0.001, η_P_^2^ = 0.15, indicating that the cognitive persistence was higher under the “10 or more ideas” condition (*M* = 2.47, *SD* = 0.78) than under the “5 ideas” condition (*M* = 2.02, *SD* = 0.22). Moreover, the interaction between creative mindset and task requirement was significant, *F*(1, 210) = 22.54, *p* < 0.001, η_P_^2^ = 0.10. Specifically, the cognitive persistence was longer under the “10 or more ideas” condition (*M* = 2.81, *SD* = 0.94) than that under the “5 ideas” condition (*M* = 1.99, *SD* = 0.18) for individuals primed for the creative growth mindset *F*(1, 213) = 49.98, *p* < 0.001, whereas there was no significant difference in the cognitive persistence between the”10 or more ideas” condition (*M* = 2.14, *SD* = 0.49) and the “5 ideas” condition (*M* = 2.04, *SD* = 0.25) for individuals primed for the creative fixed mindset, *F*(1, 213) = 0.66, *p* = 0.42. In the “10 or more ideas” condition, the cognitive persistence of individuals primed for the creative growth mindset (*M* = 2.81, *SD* = 0.94) was significantly higher than that for the creative fixed mindset (*M* = 2.14, *SD* = 0.49), *F*(1, 213) = 29.15, *p* < 0.001, whereas in the “5 ideas” condition, there was no significant difference between the two groups (creative growth mindset: *M* = 1.99, *SD* = 0.18; creative fixed mindset: *M* = 2.04, *SD* = 0.25, *F*(1, 213) = 0.12, *p* = 0.73).

#### 3.2.4. The Moderated Mediating Effect

This study aims to further investigate the mediating effect of cognitive persistence on the relationship between metacognitive (dis)fluency experiences and AUT performance. Additionally, it examines the moderating role of creative mindsets within this mediation pathway. A moderated mediation model (Model 7, Hayes 2013) was used to test our hypothesis. The result showed that metacognitive fluency experience was negatively related to cognitive persistence (*β* = −0.62, *p* < .05). Furthermore, metacognitive fluency experience was negatively related to AUT performance (*β* = −0.13, *p* < .05), while cognitive persistence was positively and significantly associated with AUT performance (*β* = 0.40, *p* < .001). Moreover, the results for the indirect effects confirm the significant mediating role of cognitive persistence in the relationship between metacognitive (dis)fluency experiences and AUT performance (indirect effect = 0.16, 95% CI with LL = 0.09 and UL = 0.24) (Table 1).

Table 1 presents the results for the conditional indirect effect of metacognitive (dis)fluency experiences on AUT performance via the cognitive persistence creative mindset. The indirect effect of metacognitive (dis)fluency experiences on AUT performance through cognitive persistence was not significant for individuals primed for the creative fixed mindset (effect = 0.10, LL 95% CI = −0.09, UL 95% CI = 0.29). However, this effect strengthened for individuals primed for the creative growth mindset (effect = 0.82, LL 95% CI = 0.59, UL 95% CI = 1.05). To further verify whether the indirect effect is affected by creative mindset, we tested whether the bootstrapped confidence interval of the moderated mediation index included zero. The results, as shown in Table 1, indicated a negative moderated mediation effect with a non-zero probability (*β* = 0.29; 95% bias-corrected CI: [0.15, 0.43]). Therefore, we conclude that creative mindsets moderate the indirect effect of metacognitive (dis)fluency experiences on AUT performance via cognitive persistence.

### 3.3. Discussion

Experiment 2 found that priming individuals with a creative growth mindset improved their cognitive persistence under the metacognitive disfluency experience condition, subsequently enhancing the creativity of the ideas generated. This suggests that creative mindsets play a moderating role in the influence of metacognitive (dis)fluency experiences on divergent thinking tasks. Similar to how individuals with different creative mindsets vary in their sensitivity to metacognitive (dis)fluency experiences and in how they use those experiences to guide idea selection during divergent thinking tasks (Betsch and Roth 2018; Puente-Díaz et al. 2021), the metacognitive disfluency experience led individuals with a creative growth mindset to pursue the cognitive persistence pathway, according to the dual pathway creativity model (De Dreu et al. 2008), thereby enhancing the creativity of their ideas. However, the gender imbalance in this experiment represents a potential limitation. Future research should aim for a more balanced gender distribution to further test these results.

## 4. General Discussion

This study found that metacognitive (dis)fluency experiences affect AUT performance, with individuals performing significantly better under the metacognitive disfluency experience condition than under the metacognitive fluency experience condition. Moreover, the moderated mediation model suggested that the indirect effect of metacognitive (dis)fluency experiences on AUT performance via cognitive persistence creative mindset, while individuals with a creative growth mindset exhibit significantly greater cognitive persistence, thereby promoting the production of creative idea under metacognitive disfluency experience condition.

We employed a retrieval fluency paradigm to restrict the number of ideas that participants were required to generate for each object (Murphy and Castel 2021; Sanna et al. 2002), directly activating their different metacognitive (dis)fluency experiences in Experiment 1. By using the average originality of ideas as the core indicator of AUT performance (Batey and Furnham 2008; Benedek et al. 2013), the results indicated that ideas generated under the metacognitive disfluency experience condition were more creative. This finding was based on the exclusion of individual motivation, which is influenced by processing fluency (Nevo et al. 2020) and affects creativity (Roskes et al. 2012). Previous studies suggested that metacognitive fluency experience induces a more intuitive processing style, whereas metacognitive disfluency experience promotes a more analytical processing style (Alter et al. 2007; Eitel et al. 2014; Kühl et al. 2014; Murphy et al. 2022; Unkelbach and Greifeneder 2013). Based on these insights, the results of Experiment 1 suggest that analytical processing could potentially enhance performance on divergent thinking tasks, particularly under specific conditions. Unsworth (2010) has noted that divergent thinking tasks often involve top-down executive control processes, and the beneficial effects of analytical processing on divergent thinking performance have been supported by prior research, both directly and indirectly (George and Zhou 2007; Mehta et al. 2012). For instance, George and Zhou (2007) demonstrated that a highly aroused emotional state can facilitate conscious, analytical, and focused processing, which may help individuals efficiently complete divergent thinking tasks.

While our findings suggest that metacognitive disfluency experiences may facilitate analytical processing, which subsequently enhances divergent thinking performance, it is crucial to recognize that this relationship is not universally consistent across all contexts. For example, empirical evidence has shown that priming analytical thinking can impair divergent creativity, as measured by the AUT (Hongdizi et al. 2023). This impairment effect may arise because analytical thinking tends to impose more constraints that restrict divergent creativity. These contrasting findings highlight the complexity of the relationship between metacognitive (dis)fluency experiences, cognitive processing styles, and creative performance. Future research should explore additional factors that may contribute to these observed effects, such as the role of individual differences, task characteristics, and the specific conditions under which metacognitive disfluency is induced.

Experiment 2 found a positive effect of creative mindset on AUT performance. Specifically, individuals with a creative growth mindset significantly outperformed those with a creative fixed mindset, regardless of whether they were under metacognitive fluency or disfluency experience conditions. Moderated mediation analysis revealed that the mediating effect of cognitive persistence on the relationship between metacognitive disfluency experience and AUT performance was significant only for individuals with a creative growth mindset. Individuals’ mindsets can interpret metacognitive disfluency experience as either insufficient effort or ability deficiency, influencing task-relevant processes, such as cognitive persistence, strategy selection, and, ultimately, final task performance (Miele and Molden 2010; Miele et al. 2013; Molden and Dweck 2006). In the current experiment, requiring participants to generate ten unusual uses for each object is inherently challenging, and differing mindsets lead to varying task goals (Dweck and Leggett 1988; Mangels et al. 2006; Müllensiefen et al. 2015). Specifically, individuals with a creative fixed mindset, driven by performance goals, may perceive the metacognitive disfluency experience as feedback indicating their inability to succeed, leading them to invest less cognitive effort and adhere to the “ease is good” heuristic. Conversely, individuals with a creative growth mindset, driven by mastery goals, believe that they can compensate for the obstacles posed by metacognitive disfluency experience through sufficient cognitive effort, adhering to the “effort is beneficial” heuristic. This highlights the contribution of cognitive persistence (i.e., prolonged analytical processing on the task) to creative performance.

**Figure 2:**
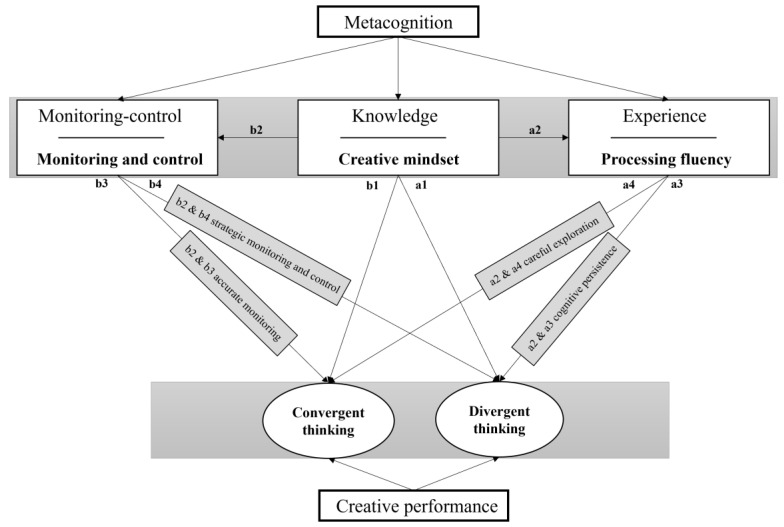
Impacts of creative mindset on divergent and convergent creative thinking processes within a metacognitive framework.

In recent years, the significance of metacognition in creativity has been increasingly acknowledged. However, relevant theories require further development, particularly regarding how metacognitive components interact to influence creative cognition (Lebuda and Benedek 2023; Preiss 2022). A systematic framework of creative metacognition proposed by Lebuda and Benedek (2023) outlines the dynamic interplay of metacognitive processes in creative tasks, where metacognitive monitoring and control depend on metacognitive knowledge, which is updated through the experience gained during creative tasks. This study aims to construct a model illustrating the impact of creative mindsets on divergent and convergent creative thinking processes within a metacognition framework (Figure 2). Serial studies suggest that creative mindsets (Karwowski 2014; O’Connor et al. 2013; Zhou et al. 2020), as crucial element of metacognitive knowledge (Jia et al. 2019; Mangels et al. 2006), influence individuals’ interpretations of metacognitive experiences (Blackwell et al. 2007; Jia et al. 2023) and the accuracy of metacognitive monitoring and control (Blackwell et al. 2007; Ehrlinger et al. 2016; Jia et al. 2022). Based on existing theoretical frameworks and research findings, we propose a model that clarifies the impact of a creative growth mindset on divergent and convergent creative thinking processes. In this model, the creative growth mindset directly impacts performance on both divergent and convergent thinking tasks (a1 and b1), consistent with previous findings (O’Connor et al. 2013; Royston and Reiter-Palmon 2019; Zhou et al. 2020). Moreover, metacognitive experiences have opposing effects on performance in these tasks (a3 and a4), supporting the dual pathway model of creativity (De Dreu et al. 2008; Nijstad et al. 2010). Importantly, the creative growth mindset moderates the relationship between metacognitive experiences and task performance in both divergent and convergent thinking. Specifically, it encourages individuals to exert greater cognitive persistence under conditions of metacognitive disfluency experience, thereby enhancing divergent thinking performance (a2 and a3). Additionally, these beliefs enable individuals to effectively mitigate the negative impact of metacognitive disfluency experience on convergent thinking tasks through careful exploration of the problem space (a2 and a4) (Ericsson and Simon 1998; Jia et al. 2023). Finally, metacognitive monitoring and control can influence creative performance (b3 and b4, De Chantal and Organisciak 2023; Gilhooly et al. 2007; Urban and Urban 2023). The creative growth mindset affects performance in divergent and convergent tasks through metacognitive (strategic) monitoring and control, facilitating the activation of top-down metacognitive strategic monitoring and control necessary for successful divergent thinking (b2 and b4, Jia et al. 2022), as well as improving performance in convergent thinking tasks through accurate metacognitive monitoring (b2 and b3). In sum, this model demonstrates that creative cognitive processes are influenced by creative mindsets, which encompass not only metacognitive experiences related to processing fluency but also other factors, such as cognitive persistence, pathway selection, strategy application, and metacognitive strategy monitoring and control, highlighting how metacognition regulates creative cognitive processes.

Practically, as growth mindset interventions have been popularized through multiple avenues, particularly in the learning area (Blackwell et al. 2007; Yeager et al. 2022), future creativity education could perhaps focus on shaping individuals’ growth-oriented creative mindset. In addition to traditional methods, such as fostering a collaborative culture (Amabile 2006), stimulating brainstorming (Paulus and Yang 2000), and nurturing intrinsic motivation (Dweck et al. 1995), it may be beneficial to deliberately encourage individuals to adopt the belief that “creativity is malleable and can be developed”. Furthermore, guiding them to engage in positive self-feedback, such as with affirmations, like “I can enhance my creativity through effort”, could represent promising avenues for cultivating their creativity.

Metacognition allows individuals to critically evaluate their cognitive processes, including information gathering, belief formation, and the application of thinking strategies. Such self-awareness fosters more flexible and adaptive approaches to creative problem-solving, a skill that is also particularly essential when addressing complex and contentious real-world issues (Fischer and Fleming 2024). For example, in the context of climate change, metacognitive skills can enable individuals and organizations to recognize the limitations of existing knowledge, thus fostering openness to new evidence and alternative perspectives (De Beukelaer et al. 2023). By cultivating metacognitive abilities, we can enhance our capacity to navigate the complexities of global challenges, mitigate cognitive biases, and promote evidence-based decision-making. Therefore, metacognition represents a vital tool for both creative problem-solving and informed action in the face of intricate, real-world problems. Future research should focus on developing metacognitive interventions tailored to different contexts and populations.

## 5. Conclusions

Overall, the two experiments demonstrated that metacognitive disfluency experience enhanced divergent thinking performance and that creative mindsets moderate the indirect effect of metacognitive disfluency experience on this performance through cognitive persistence. Specifically, metacognitive disfluency experience led individuals with a creative growth mindset to adopt the cognitive persistence pathway, thereby improving the creativity of their ideas, providing evidence for the dual pathway creativity model. A theorical model was introduced to illustrate the role of creative mindsets in creative thinking processes within a metacognitive framework. Given the complex relationship between metacognition and creativity, further research is needed to explore how these metacognitive components interact to influence creative cognition.

## Figures and Tables

**Table 1:** Conditional process analysis.

	Cofficient	*SE*	LICI	ULCI
**Mediator variable model (cognitive persistence)**				
Constant	2.72	0.36	2.00	3.44
Metacognitive experiences “cognitive persistence”	−0.62	0.23	−1.07	−0.18
Creative mindset “cognitive persistence”	−0.77	0.25	−1.26	−0.29
Metacognitive experiences × creative mindset “cognitive persistence”	0.72	0.15	0.26	1.02
**Dependent Variable Model**				
Constant	2.05	0.10	1.84	2.25
Metacognitive experiences “AUT performance”	−0.13	0.05	−0.23	−0.03
Cognitive persistence “AUT performance”	0.40	0.04	0.32	0.49
Indirect effect model				
Metacognitive experiences “cognitive persistence” AUT performance	0.16	0.04	0.09	0.24
**Conditional direct effect analysis**	Effect	Boot *SE*	BootLLCI	BootULCI
Mediator				
Creative fixed mindset	0.10	0.10	−0.09	0.29
Creative growth mindset	0.82	0.12	0.59	1.05
Index of moderated mediation	0.29	0.07	0.15	0.43

## Data Availability

The data are currently not publicly available due to participant privacy, but they are available from the first author upon reasonable request.
